# Supplementary material for: An Experimentally Determined Evolutionary Model Dramatically Improves Phylogenetic Fit
Source: Mol Biol Evol. 2014 May 24;31(8):1956–78. doi: 10.1093/molbev/msu173 (PMC4104320; doi:10.1093/molbev/msu173)
Supplement: Supplementary Data [file supp_31_8_1956__index.html]

An experimentally determined evolutionary model dramatically improves phylogenetic fit — An Experimentally Determined Evolutionary Model Dramatically Improves Phylogenetic Fit — An Experimentally Determined Evolutionary Model Dramatically Improves Phylogenetic Fit — Supplementary Data 

# An Experimentally Determined Evolutionary Model Dramatically Improves Phylogenetic Fit

## Supplementary Data

files

**Files in this Data Supplement:**

- Supplementary Data - pdf file
- Supplementary Data - pdf file
- Supplementary Data - xls file
- Supplementary Data - xls file
