# Supplementary material for: An Experimentally Determined Evolutionary Model Dramatically Improves Phylogenetic Fit
Source: Mol Biol Evol. 2014 May 24;31(8):1956–78. doi: 10.1093/molbev/msu173 (PMC4104320; doi:10.1093/molbev/msu173)
Supplement: Supplementary Data [file supp_msu173_Supplementary_figure_1.pdf]

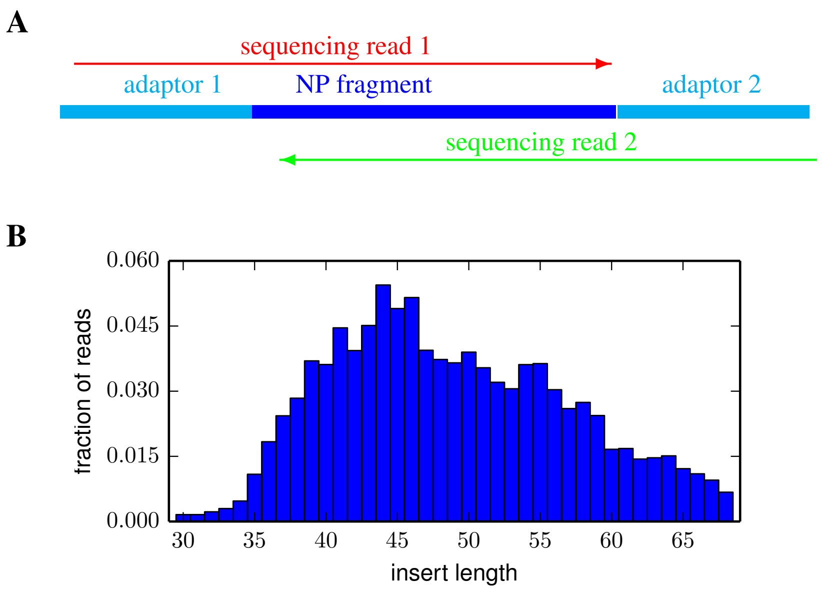


**Supplementary figure 1:** Illumina sequencing accuracy was increased by using overlapping paired-end reads. **(A)** The strategy is to shear the NP fragments to about 50 nucleotides in length, and then sequence with overlapping paired-end reads. This provides double coverage, and only codon identities for which both reads agree are called. **(B)** The actual length distribution of alignable paired reads that had at least 30 nucleotides of overlap (lengths between 30 and 70 nucleotides) for a typical sample.
