# Supplementary material for: An Experimentally Determined Evolutionary Model Dramatically Improves Phylogenetic Fit
Source: Mol Biol Evol. 2014 May 24;31(8):1956–78. doi: 10.1093/molbev/msu173 (PMC4104320; doi:10.1093/molbev/msu173)
Supplement: Supplementary Data [file supp_msu173_Supplementary_figure_2.pdf]

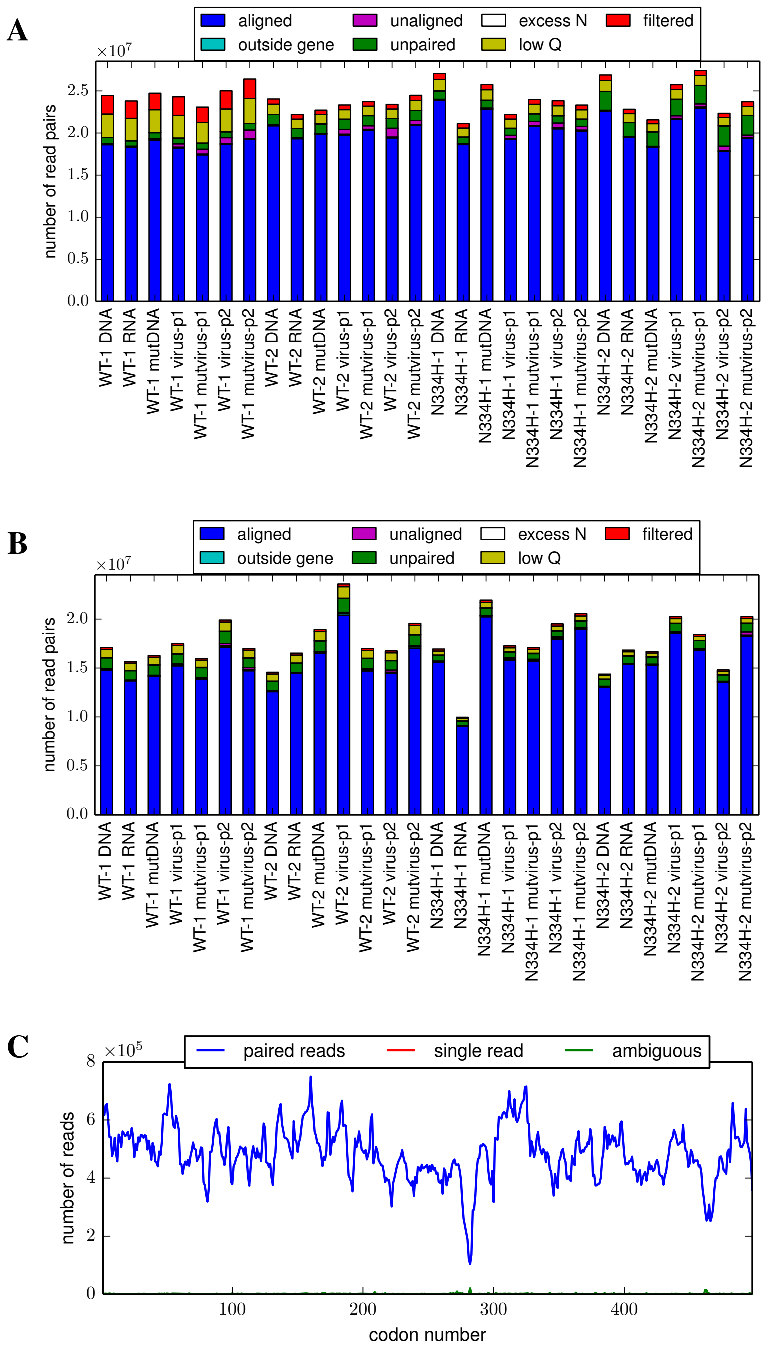

Supplementary figure 2: The number of alignable paired reads for each sample from **(A)** *replicate A* and **(B)** *replicate B*. Most read pairs passed the various filters, could be overlapped, and could have their overlap aligned to NP. **(C)** The read depth across the primary sequence for a typical sample. The read depth was not entirely uniform along the primary sequence, probably due to biases in fragmentation locations. The computer code used to generate these figures is available at http://jbloom.github.io/mapmuts/example_2013Analysis_Influenza_NP_Aichi68.html.
